# “Real-World Study of a Dual-Layer Micromesh Stent in Elective Treatment of Symptomatic and Asymptomatic Carotid Artery Stenosis (ROADSAVER)”

**DOI:** 10.1007/s00270-021-03051-5

**Published:** 2022-01-18

**Authors:** Sasko Kedev, Stefan Müller-Hülsbeck, Ralf Langhoff

**Affiliations:** 1 Cardiology Department, Medical Faculty, University St Cyril and Methodius, University Clinic of Cardiology, Vodnjanska 17, 1000 Skopje, North Macedonia; 2 Department of Diagnostic and Interventional Radiology/Neuroradiology, Academic Hospitals Flensburg, Flensburg, Germany; 3 Department for Angiology, Center for Internal Medicine I, Brandenburg Medical School Theodor Fontane, Campus Clinic Brandenburg, Brandenburg an der Havel, Germany

**Keywords:** Roadsaver, Dual-layer micromesh stent, Carotid artery disease, Carotid artery stenting, Stroke

## Abstract

**Purpose:**

Endovascular carotid artery stenosis treatment is associated with a higher peri- and early post-procedural stroke risk relative to surgery. Dual-layer micromesh carotid stents were specifically designed for improved plaque coverage to reduce the cerebral embolization risk and related ischemic events. ROADSAVER study aims to further confirm the safety and efficacy of the Roadsaver™ dual-layer micromesh stent for the treatment of elective patients with carotid artery stenosis.

**Materials and Methods:**

ROADSAVER is a prospective, multi-center, observational study. Between January 2018 and February 2021, a total of 1967 patients featuring asymptomatic or symptomatic, non-occlusive and non-thrombotic carotid stenosis eligible for an elective stenting procedure were enrolled across 13 European countries (52 centers). Follow-up visits are scheduled at 30 days and at 12 months. The primary outcome measure is the major adverse event rate, i.e., cumulative incidence of any death or stroke up to 30 days post-procedure. All deaths, strokes and carotid revascularizations are adjudicated by an independent Clinical Events Committee. Sub-analyses are prespecified and focused on baseline patient characteristics (e.g., age, neurologic status), procedural features (e.g., access route, embolic protection use), advanced imaging, and treatment efficacy up to 12 months.

**Conclusion:**

The present study evaluates the Roadsaver™ dual-layer micromesh carotid stent in the real-world clinical practice aiming to provide valuable insights into the contemporary European treatment trends and outcomes of elective carotid artery stenting. The large study population and predefined sub-analyses should help identify the best practices and patient subsets to benefit most from the treatment.

****Trial Registration**:**

Clinicaltrial.gov identifier: NCT03504228.

## Introduction

Stroke is one of the leading causes of disability and morbidity in the western world. With more than 80 million survivors in 2016 globally, it represents a substantial socio-economic burden [1]. Carotid artery disease is directly responsible for 10–15% of all ischemic strokes [2]. The use of medical therapies and lifestyle modifications limits the associated risk factors. However, in patients with high grade stenosis and/or accompanying neurological symptoms, surgical carotid endarterectomy (CEA) or percutaneous carotid artery stenting (CAS) is indicated.

The growing CAS expertise, better patient selection, lesion-tailored strategies and evermore advanced techniques and devices continuously improve CAS outcomes. The latest European guidelines recommend CAS in patients at high risk for surgery [3]. While CAS provides a minimally invasive alternative to CEA, the risk of peri- and early post-procedural distal embolization events with potential neurological sequalae warrants further attention.

The impact of different stent designs on CAS outcomes remains debated. With conventional single layer carotid stents, particularly those featuring open-cell design and large free-cell area, the plaque prolapse through the stent struts has been associated with cerebral embolization risk and related ischemic events [4]. Dual-layer micromesh stent(s) (DLMS) were specifically designed for improved lesion coverage and prevention of plaque prolapse through the stent struts, to minimize the ischemic events during and after CAS.

The use of DLMS is associated with a low rate of major adverse events, and good mid-to-long-term treatment outcomes [5–7]. Advanced imaging studies confirm good lesion scaffolding properties and plaque prolapse limiting capacity of DLMS [8–11]. A randomized study shows that the use of DLMS in combination with proximal embolic protection lowers the number of cerebral micro-emboli detected during CAS relative to a single-layer carotid stent [12]. In contemporary clinical practice, DLMS are preferentially used for CAS treatment of high-risk patients/lesions with good outcomes [13]. While DLMS are anticipated to become a new standard in CAS [14], more “real-world” evidence from broader, unselected populations is needed to validate their clinical performance. The ROADSAVER study aims to confirm the safety and efficacy of the Roadsaver™ DLMS in a large patient cohort undergoing elective carotid artery stenosis treatment.

## Materials

**Fig. 1:**
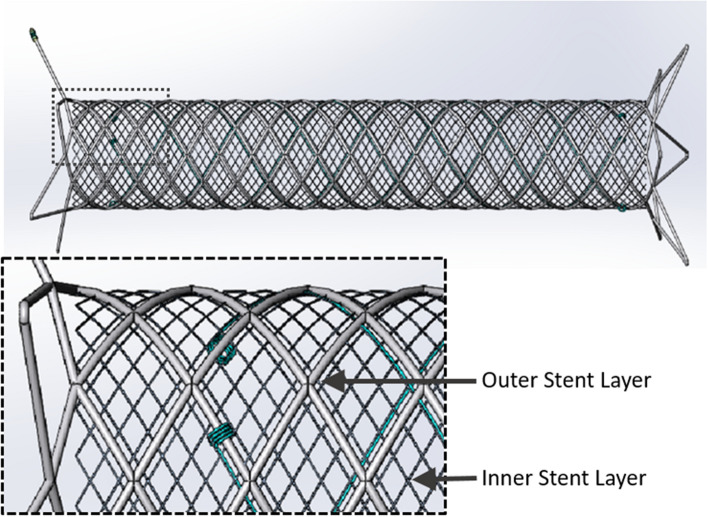
Roadsaver™ dual-layer micromesh stent (DLMS) design. (**Inset**) The stent outer layer is comprised of a braided closed cell structure with flared ends, while the inner layer consists of a braided micromesh with 375–700 µm sized pores

The Roadsaver™ DLMS system (Terumo Corporation, Tokyo, Japan) consists of a self-expanding stent and a 5-Fr rapid exchange delivery catheter of low crossing profile (1.7 mm diameter). The stent is built of nickel-titanium (nitinol) alloy and has a dual-layer braided design. The outer layer is comprised of a flexible and conformable closed cell structure with flared ends, while the inner layer forms a micromesh with 375–700 µm-sized pores (Fig. 1). The stent (outer) diameter ranges between 5–10 mm. The (unconstrained) length of the plaque-covering dual-layer varies between 16–40 mm (22–47 mm including flares). The stent is re-sheathable up to 50% deployment, allowing repositioning. The delivery system is 0.014″-guidewire-compatible and 143 cm-long.

## Methods

### Study Design & Population

ROADSAVER is a prospective, multi-center, observational study. The patient enrollment took place between January 2018 and February 2021. A total of 1967 patients featuring asymptomatic or symptomatic carotid artery stenosis were selected across 13 European countries (52 sites), including Hungary (7), Germany (12), Belgium (5), Spain (11), Poland (3), North Macedonia (2), France (2), Slovakia (2), Portugal (1), Czech Republic (2), Latvia (1), Netherlands (2) and Serbia (2). Study population consists of patients considered eligible for elective CAS as per routine hospital practice, and includes males and females derived from general interventional radiology or angiology populations who met the selection criteria (Table 1). A patient is considered to be enrolled after a successful guidewire passage through the study target lesion. The study is sponsored by Terumo Europe.

**Table 1:** Patient selection criteria

*Inclusion criteria*
The patient has a non-occlusive and non-thrombotic carotid artery stenosis
eligible to be treated with Roadsaver™ Carotid Stent as per the Instructions for Use
At least 18 years of age
Life expectancy of at least 12 months from the date of the index procedure
Informed consent signed
*Exclusion criteria*
Any condition that makes patient unsuitable for percutaneous transluminal angioplasty, including intolerance or allergy to any material used and accompanying therapy

### Procedure

Baseline evaluations, including diagnostic imaging, along with the eventual CAS procedure itself were performed according to the hospitals’ routine practice. This includes administration of the appropriate anticoagulation regimen and other therapies, e.g., treatment of vasospasm, hypotension and arrhythmias. The use of pre-/post-dilatation, or any other devices (e.g., embolic protection) was performed at operator’s discretion, much like the prescription of appropriate post-procedural antithrombotic therapy. Lesions were assessed angiographically pre- and post-procedure to quantify the degree of stenosis.

Baseline patient, lesion and procedure characteristics, concomitant medication, adverse events and follow-up data are recorded in the validated electronic case report form continuously, throughout the study duration. Follow-up assessments are scheduled at 30 days (± 7 days) and at 12 months (± 30 days). If hospital’s routine practice, a neurological examination (NIHSS: National Institutes of Health Stroke Scale) and a duplex ultrasound evaluation to assess carotid artery patency are performed. At sites where diffusion weighted magnetic resonance imaging (DW-MRI) is done standardly, the advanced cerebral imaging complements other baseline, post-procedural and 30-day follow-up assessments.

### Primary Outcome Measure

The primary outcome measure of the present study is the rate of major adverse events (MAE), i.e., the cumulative incidence of any death or stroke, up to 30 days after the index procedure, where stroke is defined as an acute neurologic event with focal symptoms and signs lasting for ≥ 24 h.

### Secondary Outcome Measures: See Table 2

**Table 2:** Secondary outcome measures

(1) Technical success: Defined as a successful access and deployment of the device with recanalization, determined by < 30% residual stenosis by angiography during the index procedure
(2) Procedural success: Defined as technical success with no device-/procedure-related death, stroke or any other serious adverse events
(3) Device malfunction: Defined as the failure of a device after its introduction into the patient (i.e., failure to perform in accordance with its intended purpose when used as per the Instructions For Use or the Clinical Investigation Plan)
(4) Death (any, stroke-related)* up to 30 days
(5) Stroke (any, major/minor)* up to 30 days *Stroke* An acute neurologic event with focal symptoms and signs, lasting for 24 h or more *Major stroke* A new neurological event that persists for > 24 h and results in a > 4 point increase in the NIHSS score relative to baseline or any subsequent lower score *Minor stroke* A new neurological event that resolves completely within 7 days or increases the NIHSS by ≤ 4 points
(6) Transient ischemic attack up to 30 days
(7) Target lesion revascularization* up to 30 days: Defined as any revascularization procedure of the original treatment site, including angioplasty, stenting, endarterectomy, or thrombolysis, performed to open or increase the luminal diameter inside or within 5 mm of the previously treated lesion
(8) Major vascular and bleeding complications up to 30-days: (a) Major hematoma, i.e., one requiring transfusion, surgical evacuation, or delay in discharge, (b) Pseudo aneurysm or arteriovenous fistula or retroperitoneal bleeding, (c) Peripheral ischemia/nerve injury caused by the proximal access site, (d) Vascular surgical repair to correct a local vascular access site complication and bleeding
*In a subgroup of patients that undergoes 12-month follow-up as per standard of care,* *the following incidence rates will be assessed*
(1) Death (any, stroke-related)* up to 12 month
(2) Stroke (any, ipsilateral)* up to 12 month *Stroke* An acute neurologic event with focal symptoms and signs, lasting for 24 h or more *Ipsilateral Stroke* Defined as a stroke occurring within the vascular distribution of the stented artery
(3) Target lesion revascularization (TLR)* up to 12 months: Defined as any revascularization procedure of the original treatment site, including angioplasty, stenting, endarterectomy, or thrombolysis, performed to open or increase the luminal diameter inside or within 5 mm of the previously treated lesion
(4) In-stent restenosis up to 12 months, measured within the stented lesion or within 5 mm proximal or distal to the stent, defined as. ≥ 50% stenosis by ultrasound (Peak Systolic Velocity Ratio (PSVR; PSV_ICA_/PSV_CCA_) > 2) or ≥ 70% stenosis by angiography)
(5) External carotid artery patency up to 12 months determined as per duplex ultrasound assessment

*All deaths, strokes and revascularizations are adjudicated by an independent clinical events committee

The ROADSAVER study also predefines a number of sub-analyses aiming to assess the impact on outcomes of different baseline patient and procedure characteristics, including age, gender, neurologic status at presentation, complex anatomy, comorbidities, access route, and embolic protection device use. Additionally, geographic- and operator specialty-related differences in practice and outcomes will be assessed. Finally, the DW-MRI imaging sub-analysis (including an independent image review) and dedicated 12-month follow-up evaluations (see Table 2), will provide further quantitative and clinically relevant insights regarding the device performance.

### Study Sample Size

Combining the results of 7 clinical trials (BEACH, CASES-PMS, SEcuRITY, CREST, EXACT, CAPTURE 2, SPACE) [15–20], a weighted mean 30-day MAE rate of 4.3% was calculated as the objective performance criterion. Using a 1.3% non-inferiority delta, 5.6% MAE rate was determined as the upper bound of the non-inferiority margin. To provide > 80% power with a one-sided significance level of 0.05, and a 7% attrition rate, a sample size of up to 2000 patients was calculated.

## Discussion

The use of conventional single layer stents in CAS has been associated with an increased incidence of cerebral ischemic events (primarily minor strokes) up to 30 days post-procedure relative to CEA [21]. DLMS including Roadsaver™ and CGuard™ (Inspire MD) were specifically designed to limit cerebral embolization and related ischemic events. The micromesh layer, featuring micron-sized pores, enables DLMS to contain the plaque debris and prevent its dislodgment, providing protection throughout the stent deployment, post-dilatation, and early post-stenting phase. With its low crossing profile, the Roadsaver™ DLMS delivery system, in addition, facilitates lesion crossing, in many cases eliminating the need for pre-dilatation. Braided (interwoven) stent design, in turn, provides good in-vessel flexibility and wall apposition in tortuous anatomies.

With the large prospective real-world patient cohort treated using a single carotid stent type, the present study aims to complement the earlier safety and efficacy data on the use of the Roadsaver™ DLMS. Numerous prespecified sub-analyses, in turn, are included to provide undistorted insights into the contemporary European CAS practice with DLMS, and to identify different treatment trends, best clinical practices, and specific patient subsets to benefit most from the elective treatment. The study also aspires to enable detection of rare events (e.g., stent thrombosis), which otherwise, in context of usually smaller in size and more controlled randomized studies, would have been missed.

## Limitations

The main limitations of the present study include observational, non-randomized design, relatively short follow-up period of 1-year, and the fact that some of the follow-up examinations, like neurologic assessment, duplex ultrasound, and DW-MRI, are not going to be available in the entire study population.

## Conclusion

The CAS field is still under critical observation, and the optimal way to limit procedural complications is not yet fully defined. More real-world clinical evidence is needed. With close to 2000 patients enrolled, the large, observational, ROADSAVER study aims to expand the knowledge on CAS using DLMS by providing valuable clinical insights. The primary study outcomes are expected in early 2022.
